# Comparison between multimodal and intraoperative opioid free anesthesia for laparoscopic sleeve gastrectomy: a prospective, randomized study

**DOI:** 10.1038/s41598-023-39856-2

**Published:** 2023-08-04

**Authors:** Piotr Mieszczański, Grzegorz Górniewski, Paweł Ziemiański, Radosław Cylke, Wojciech Lisik, Janusz Trzebicki

**Affiliations:** 1 1st Department of Anesthesiology and Intensive Care, Medical University of Warsaw, Warszawa, Poland; 2 Szpital Kliniczny Dzieciątka Jezus, ul. Lindleya 4, 02-005 Warszawa, Poland; 3 Department of General Surgery and Transplantology, Medical University of Warsaw, Warszawa, Poland

**Keywords:** Gastroenterology, Endocrine system and metabolic diseases, Gastrointestinal diseases, Metabolic disorders, Nutrition disorders

## Abstract

Anesthesia for laparoscopic sleeve gastrectomy and perioperative management remains a challenge. Several clinical studies indicate that opioid-free anesthesia (OFA) may be beneficial, but there is no consensus on the most optimal anesthesia technique in clinical practice. The aim of our study was to assess the potential benefits and risks of intraoperative OFA compared to multimodal analgesia (MMA) with remifentanil infusion. In a prospective, randomized study, we analyzed 59 patients’ data. Primary outcome measures were oxycodone consumption and reported pain scores (numerical rating scale, NRS) at 1, 6, 12, and 24th hours after surgery. Postoperative sedation on the Ramsay scale, nausea and vomiting on the PONV impact scale, desaturation episodes, pruritus, hemodynamic parameters, and hospital stay duration were also documented and compared. There were no significant differences in NRS scores or total 24-h oxycodone requirements. In the first postoperative hour, OFA group patients needed an average of 4.6 mg of oxycodone while the MMA group 7.72 mg (*p* = 0.008, *p* < 0.05 statistically significant). The PONV impact scale was significantly lower in the OFA group only in the first hour after the operation (*p* = 0.006). Patients in the OFA group required higher doses of ephedrine 23.67 versus 15.69 mg (*p* = 0.039) and more intravenous fluids 1160 versus 925.86 ml (*p* = 0.007). The mode of anesthesia did not affect the pain scores or the total dose of oxycodone in the first 24 postoperative hours. Only in the first postoperative hour were an opioid-sparing effect and reduction of PONV incidence seen in the OFA group when compared with remifentanil-based anesthesia. However, patients in the OFA group showed significantly greater hemodynamic lability necessitating higher vasopressor doses and more fluid volume.

## Introduction

Patients with obesity undergoing bariatric surgery, including the most commonly performed laparoscopic sleeve gastrectomy (LSG), are particularly vulnerable to opioid side effects such as respiratory depression, postoperative nausea, and vomiting (PONV) as well as excessive sedation^1–3^. To reduce opioid use, the Enhanced Recovery After Bariatric Surgery (ERABS) guidelines recommend multimodal analgesia, such as the administration of co-analgesics, regional anesthesia, or non-opioid analgesics^3,4^. These agents in combination make it possible to eliminate the intraoperative use of opioids, which is referred to as opioid-free anesthesia (OFA)^3^.

While opioid-free anesthesia (OFA) has shown potential benefits, it is not without risks. In order to address concerns surrounding efficacy and safety following laparoscopic sleeve gastrectomy (LSG) surgery^5,6^, a prospective, randomized, single-blind study was conducted. The study aimed to compare anesthetic techniques utilizing multimodal analgesia with remifentanil to intraoperative OFA to provide objective data to assist in decision-making and help balance these techniques' potential risks and benefits.

## Materials/methods

The study was conducted by the 1st Department of Anesthesiology and Intensive Care, Medical University of Warsaw, Poland. Study participants were recruited among patients qualified for elective LSG in the Department of General Surgery and Transplantology between February 2020 and October 2022. Approval for the study was granted by the Bioethics Committee of the Medical University of Warsaw (KR/5/2020), and the study was registered on 07.02.2020 with clinicaltrials.gov (NCT04260659). The study was compliant with the principles outlined in the Declaration of Helsinki, and the manuscript adheres to the applicable CONSORT guidelines.

### Study design

The study was designed as a single-blind, randomized, controlled trial. Equal, parallel 1:1 randomization was performed using http://www.randomization.com (Dallal GE). The list was generated on 08.02.2020 and accessed by only one investigator, who informed the anesthesiologist of group eligibility one hour preoperatively.

A sample size of 60 patients was calculated based on the Altman nomogram to obtain a 30% reduction in postoperative opioid consumption with a significance and power of 90%^2^.

The patient, the surgical team, and the Post-Anesthesia Care Unit (PACU) staff remained blinded.

Patients scheduled for surgery had a BMI > 40 or > 35 but with comorbidities, were aged 18 to 65, and were LSG-eligible. Informed, written consent was obtained from all participants by one of the investigators. Patients who did not consent to participation in the study, were undergoing revision surgery, had an allergy to any of the drugs used in the protocol, and were unable to cooperate in assessing pain intensity on the numerical rating scale (NRS) scale or use patient-controlled analgesia (PCA) pump were excluded from the study. After the randomization, we excluded from the analysis patients with a change in the extent of surgery.

Patients underwent standardized preoperative preparation. All were instructed preoperatively on how to use a PCA pump and rate pain using the NRS scale.

Anesthesia was conducted according to a protocol based on ESRA Prospect recommendations^7^. One hour before surgery, all patients received paracetamol 1 g i.v., metamizole 2.5 g i.v. and dexamethasone 8 mg i.v. Induction was performed using propofol 2–2.5 mg/kg, while anesthesia was maintained with desflurane. Bispectral Index (BIS) was utilized to monitor awareness with a target value of 40–60. Local infiltration of the trocar insertion sites with 0.25% bupivacaine (40 ml total) was performed by the surgeon intraoperatively.

Before induction, patients in the OFA group received a 10-min infusion of dexmedetomidine (1 mcg/kg ideal body weight—IBW) and lidocaine (1.5 mg/kg IBW). IBW was defined according to Brock formula. In the OFA group, ketamine 0.5 mg/kg IBW i.v. was also administered immediately after propofol. After endotracheal intubation, continuous infusion of dexmedetomidine and lidocaine was started with a dose depending on hemodynamic parameters toa maximum of 1 mcg/kg IBW/h and 3 mg/kg IBW/h, respectively, with a stable solution of dexmedetomidine 100mcg and lidocaine 300 mg with 0.9% NaCl to 20 ml in one syringe^8^. OFA group also received 40–50 mg/kg of magnesium sulfate IBW in balanced fluid solution i.v. If tachycardia above 120/minute with concomitant hypertension above 140/90 mmHg occurred, rescue fentanyl 100 mcg i.v. was to be given.

In the multimodal analgesia (MMA) group, remifentanil in a 2 mg/40 ml solution was dosed using the Target Controlled Infusion (TCI) pump according to the Minto model. The pump was programmed with an IBW set as patient weight and target plasma concentration for induction of anesthesia set to 6 ng/ml^9^. The maintenance dose was adjusted depending on hemodynamic parameters.

In case of bradycardia < 48/min, atropine 0.5 mg was administered and if MAP dropped < 60 mmHg, ephedrine up to a maximum dose of 50 mg in both groups was administered. If, despite that, MAP persisted below 60 mmHg, norepinephrine infusion was started. In our center, after the resection was completed, surgeons asked for a systolic pressure > 120 mmHg to check for hemostasis.

Muscle relaxation was achieved initially by administration of succinylcholine 1–1.5 mg/kg i.v., followed by rocuronium or cis-atracurium to achieve Train of Four (TOF) < 1 during surgery. Residual effects of muscle relaxants were reversed by sugammadex or neostigmine with atropine under TOF control. The decision to choose a muscle relaxant depended on the anesthesiologist’s decision and the availability of sugammadex.

Intraoperative heart rate (HR), systolic and diastolic blood pressure (BP) were measured invasively after radial artery cannulation, and pulse oximetry was monitored. Ventilation was managed to achieve an end-tidal carbon dioxide (EtCO2) of 35–45 mmHg and SpO2 > 94%.

After wound closure, dexmedetomidine and lidocaine in the OFA group or remifentanil in the MMA group were discontinued, and oxycodone was administered at a dose of 0.1 mg/kg IBW i.v.

Following extubation, patients were transported to the PACU, where analgesic treatment was administered based on paracetamol 1 g i.v., metamizole 1 g i.v. given every 6 h, and oxycodone (bolus 2 mg, lockout 10 min) administered via a PCA iv pump. All patients in the PACU received 5 l/min oxygen therapy for the first 2 h. In case of nausea, a single dose of ondansetron 4 mg i.v. was administered. Patients remained in the PACU for 24 h after surgery; thereafter they were discharged home.

### Primary and secondary outcome measures

The primary outcome measures were total oxycodone consumption and pain scores on the NRS scale 1,6,12 and 24 h after surgery. Parameters such as postoperative sedation on the Ramsay scale, PONV impact scale^10^, desaturation episodes < 94%, pruritus 1, 6, 12 and 24 h after surgery, highest and lowest intraoperative HR and BP, as well as MAP, were also documented. Further outcome measures were total fluid volume, total ephedrine dose, the need to use norepinephrine infusion or rescue fentanyl in the OFA group, operative and anesthesia time, time to extubation and the ability to discharge the patient home 24 h after surgery.

Statistical analysis was performed using the Statistica 13.1 package (TIBCO Software Inc. (2017). Statistica (data analysis software system), version 13. http://statistica.io.Dell Inc.). Even continuous variables due to low group sizes and deviating from normal (Shapiro–Wilk test) or asymmetric distributions were analyzed using non-parametric tests (Mann–Whitney U). Nominal and ordinal variables were analyzed using the Chi2 test, with Yates correction when indicated that is for expected counts below 10. *P*-value < 0.05 was considered statistically significant.

## Results

### Study population

**Figure 1:**
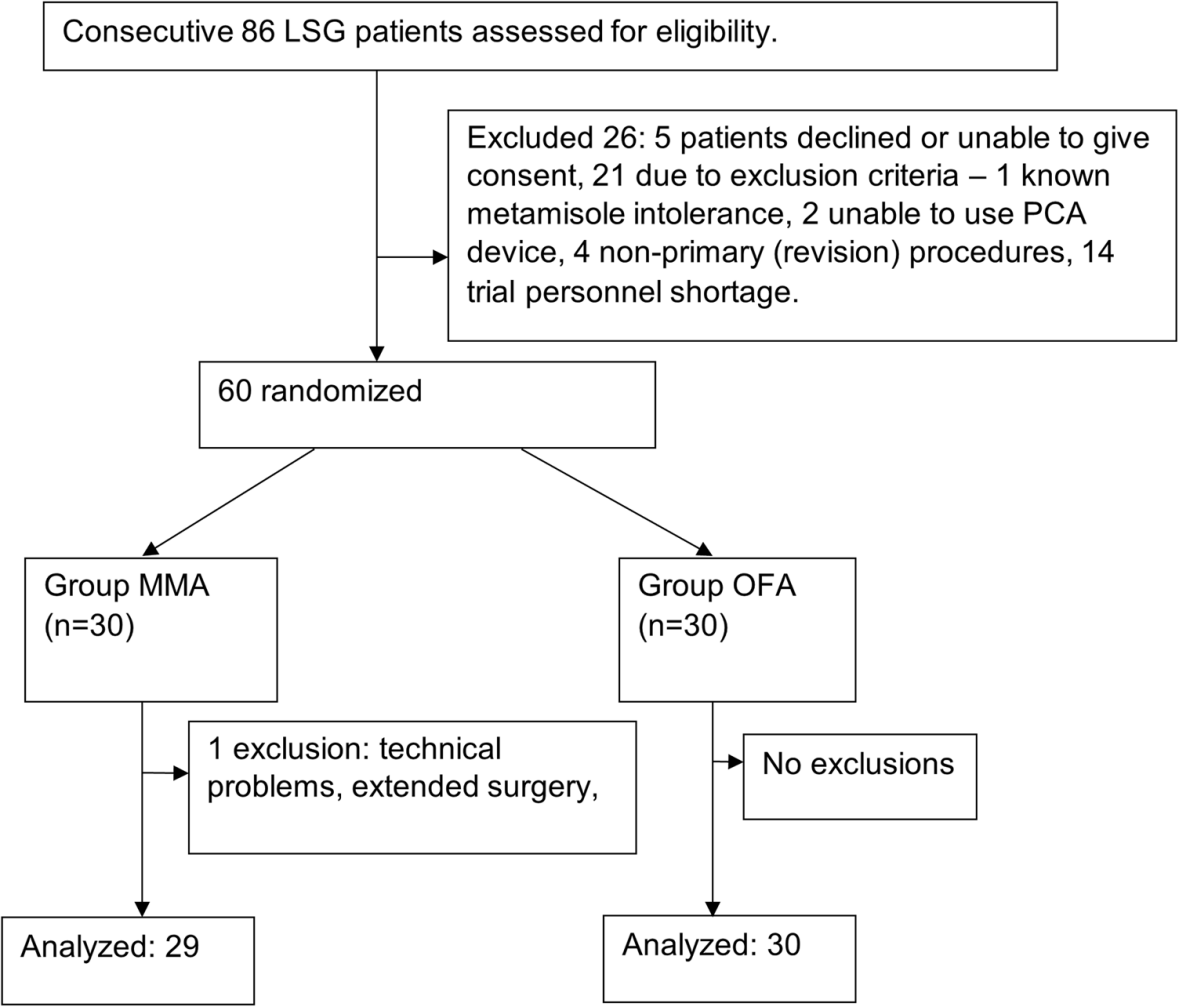
Study chart flow.

A total of 60 patients were eligible for the study, and one patient was excluded from the analysis due to suspected bowel injury resulting in much-elongated surgery time. Finally, 30 patients were included in the OFA group and 29 in the MMA group (Fig. 1). There were no significant differences between the groups in relation to the distribution of age, sex, BMI, duration of anesthesia, the procedure itself, or time from the end of the surgical procedure to extubation (Table 1). *P*-value < 0.05 was considered significant.

**Table 1:** Selected characteristics of the study population.

Variable	All patients	OFA group	MMA group	*p*-value (Mann–Whitney U test) *p* < 0.05 statistically significant
	Mean (SD)			
Age (y)	43.92 (10)	45.1 (11.6)	42.73 (8.1)	0.13
Weight (kg)	130.75 (23.1)	130.81 (23.6)	130.69 (23)	0.9
Ideal body weight—IBW (kg)	67.10 (12.1)	67.13 (12.6)	67.07 (11.8)	0.83
Height (m)	1.70 (0.1)	1.69 (0.1)	1.71 (0.1)	0.9
BMI	45.16 (7.2)	45.42 (7.9)	44.9 (6.5)	0.85
Anesthesia duration (min)	108.15 (24.5)	108.9 (26.5)	107.38 (22.8)	0.86
Surgery duration (min)	87.81 (26.3)	86.27 (27.4)	89.41 (25.5)	0.5
Time to extubation (min)	8.8 (8.9)	8.77 (3.9)	8.83 (12.2)	0.09

### Intraoperative management

There was no significant difference between the groups in the type of drug used to reverse skeletal muscle relaxation (neostigmine, sugammadex). Patients in the MMA group were not significantly more likely to require atropine to treat bradycardia (3 patients in the OFA group vs. 4 patients in the MMA group, *p* = 0.49, Chi^2^).

There was a significant difference in the volume of administered intravenous fluids between the groups—on average, 234.14 ml more fluids were required by patients in the OFA group. They also needed a significantly higher dose of ephedrine. No significant differences were observed regarding minimal and maximal BP and maximal HR values. However, a significantly lower value of the lowest observed HR in the MMA group was measured (Table 2). In 2 patients in the OFA group, a rescue dose of fentanyl was required due to tachycardia/and hypertension, even though the BIS value was below 60. In 2 patients in each group, norepinephrine infusion was necessary.

**Table 2:** Selected intraoperative parameters—values of blood pressure and heart rate, total volume of administered intraoperative i.v. fluids administered and total ephedrine dose.

Variable	All patients	OFA group	MMA group	*p*-value (Mann–Whitney U test) *p* < 0.05 statistically significant
	Mean(SD)	Mean (SD)	Mean (SD)	
Intraoperative i.v. fluids volume (ml)	1044.91 (345.38)	1160 (32.85)	925.86 (326.95)	0.007
Intraoperative ephedrine dose (mg)	19.75 (14.4)	23.67 (14.2)	15.69 (13.68)	0.039
Max heart rate (1/min)	90.93 (12.27)	91.3 (12.30)	90.55 (12.45)	0.710
Max systolic blood pressure (mmHg)	134.54 (21.18)	135.9 (23.63)	133.13 (18.62)	0.946
Max diastolic blood pressure (mmHg)	78.41 (14.48)	82.03 (15.13)	74.65 (12.97)	0.105
Max mean blood pressure—(mmHg)	97.12 (16.11)	99.99 (17.54)	94.14 (14.17)	0.262
Min heart rate—HR (1/min)	64.90 (11.50)	68.7 (10.95)	60.96 (10.87)	0.019
Min systolic blood pressure—SAP (mmHg)	81.15 (11.15)	79.47 (9.62)	82.89 (12.48)	0.234
Min diastolic blood pressure—DAP (mmHg)	48.88 (7.51)	48.77 (6.73)	49 (8.36)	0.988
Min. mean blood pressure (mmHg)	59.64 (8.25)	59 (7.23)	60.29 (9.28)	0.644

### Opioid consumption and NRS values

There were no significant differences in reported pain (NRS) or total oxycodone requirements between the groups (Table 3). However, at 1 h after the procedure, the dose of oxycodone administered via the PCA pump was significantly lower in the OFA group compared to the MMA group, 4.6 mg of oxycodone (SD 4.34) versus 7.72 mg (SD 4.56) respectively. There were no significant differences at the other assessment intervals (Table 4).

**Table 3:** Comparison of reported pain score values at consecutive time points (NRS scale) and cumulative postoperative oxycodone dose.

Variable	All patients	OFA group	MMA group	*p*-value (Mann–Whitney U test) *p* < 0.05 statistically significant
	Mean (SD)			
NRS 1 h	3.81 (2.84)	3.57 (3.05)	4.06 (2.64)	0.524
NRS 6 h	2.37 (1.83)	2.27 (1.87)	2.48 (1.81)	0.666
NRS 12 h	2.46 (1.96)	2.45 (2.28)	2.46 (1.62)	0.632
NRS 24 h	2.68 (2.48)	2.93 (2.70)	2.41 (2.24)	0.601
Oxycodone cumulative PACU dose [mg]	23.62 (14.04)	24.41 (13.64)	22.83 (14.62)	0.405
Oxycodone cumulative total dose [mg]	30.31 (14.07)	31.31 (13.70)	29.31 (14.6)	0.380

**Table 4:** Comparison of the cumulative dose of oxycodone administered from the PCA pump at consecutive time points.

Variable	All patients	OFA group	MMA group	*p*-value (Mann–Whitney U test) *p* < 0.05 statistically significant
	Mean (SD)			
Oxycodone 1 h (mg)	6.14 (4.68)	4.6 (4.34)	7.72 (4.56)	0.008
Oxycodone 6 h (mg)	14.0 (9.05)	13.6 (9.63)	14.41 (8.56)	0.603
Oxycodone 12 h (mg)	18.62 (11.71)	18.48 (11.2)	18.76 (12.39)	0.914
Oxycodone 24 h (mg)	23.66 (14.07)	24.48 (13.7)	22.83 (14.62)	0.395

The difference at 1 h is statistically significant (Mann–Whitney U test, *p* = 0.008).

The two patients requiring a rescue dose of fentanyl did not impact our results. Within 24 h after the surgery, they received 40 and 48 mg of oxycodone, respectively, whereas the maximal dose for the whole study population was 65 mg and 54 mg for the OFA group. These two patients also had no impact on other analyses, including the side effects profile.

### Oxycodone side effects and hospital stay duration

The PONV impact scale differed significantly between groups only in the first hour after surgery (Mann–Whitney U test, *p* = 0.006). (Table 5). There were no significant differences in the incidence of desaturation < 94% or in the assessment of pruritus between the groups (Table 6.). In the OFA group, 8 patients were not discharged from the hospital the following day after the procedure, compared to 2 patients in the MMA group. However, the difference was not statistically significant. In addition, only in 3 patients (all in the OFA group) was this delay directly related to postoperative pain management. These patients were discharged on the following day.

**Table 5:** Comparison of the incidence of nausea and vomiting (PONV Impact score) at 1,6,12 and 24 h after surgery.

Variable	All patients	OFA group	MMA group	*p*-value (Mann–Whitney U test) *p* < 0.05 statistically significant
	Median (IQR)	Median (IQR)	Median (IQR)	
PONV Impact 1 h	0 (2)	0 (0)	1 (2)	0.006
PONV Impact 6 h	0 (1)	0.5 (1)	0 (2)	0.66
PONV Impact 12 h	0 (1)	0 (1)	0 (1)	1
PONV Impact 24 h	0 (1)	0 (1)	0 (1)	0.67

**Table 6:** Incidence of desaturation < 94% and pruritus at 1,6,12 and 24 h after surgery.

Variable	All patients		OFA group		MMA group		*p*-value (Chi^2^) *p* < 0.05 statistically significant
	No	Yes	No	Yes	No	Yes	
SaO2 < 94% after 1 h	45 (76.27%)	14 (23.73%)	21 (70%)	9 (30%)	24 (82.76%)	5 (17.24%)	0.25
SaO2 < 94% after 6 h	57 (96.61%)	1 (1.69%)	30 (100%)	0	28 (96.55%)	1 (3.45%)	0.99
SaO2 < 94% after 12 h	54 (91.53%)	2 (3.39%)	30 (100%)	0	27 (93.5%)	2 (6.9%)	0.46
SaO2 < 94% after 24 h	58 (98.31%)	0 (0%)	30 (100%)	0	29 (100%)	0	
Pruritus 1 h	55 (93.22%)	2 (3.39%)	30 (100%)	0	27 (93.1%)	2 (6.9%)	0.46
Pruritus 6 h	55 (93.22%)	2 (3.39%)	30 (100%)	0	27 (93.1%)	2 (6.9%)	0.46
Pruritus 12 h	55 (93.22%)	2 (3.39%)	29 (96.67%)	1 (3.33%)	28 (96.55%)	1 (3.45%)	1
Pruritus 24 h	49 (83.05%)	8 (13.56%)	25 (83.33%)	5 (16.67%)	26 (89.66%)	3 (10.34%)	0.74
Hospital discharge within 24 h	10 (16.95%)	49 (83.05%)	8 (26.67%)	22 (73.33%)	2 (6.9%)	27 (93.1%)	0.094

## Discussion

Our prospective, randomized controlled trial found that OFA during the surgery did not affect total postoperative opioid consumption or NRS score. Additionally, the reduction of incidence of PONV and opioid consumption was demonstrated only in the immediate postoperative care setting, and this effect did not persist for six or more hours. Patients in the OFA group were hemodynamically more labile and required more vasopressor support and fluid volume.

There are controversies in the evaluation of the potential benefits of OFA. Our study aligns with previous research^2,11–14^ and a bariatric surgery meta-analysis^15^, showing no significant difference in total postoperative opioid consumption. Similar to our results, in a study by Mulier 2018^2^, the reduction of NRS score and the opioid dose was significant only a few hours after the operation and not later^2^. On the contrary, several trials revealed the superiority of OFA in this aspect. Ubing et al.^16^ found less opioid use in 48 h after the operation with significantly lower pain scores. The main difference that could contribute to these results is the continuous administration of the coanalgesics mixture in the recovery room, which may prolong the effects observed in our study only one hour after the operation. Ibrahim et al. demonstrated less morphine use with lower NRS in the first 6 h^17^. Still, in their study, both groups had bilateral subcostal transversus abdominis plane blocks, an essential factor that may impact this result. Reduced total opioid requirement with associated improved pain scores was also seen in recent studies by Ahmed and Soudi et al.^18,19^. The latter trial observed lower pain scores throughout 24 h after the operation. This might result from a relatively higher dose of dexmedetomidine, which was 1 mcg per kilogram of total and not ideal body weight as in our study.

In a broader context, a 2021 meta-analysis by Salome et al. ^20^ that included 33 studies on patients undergoing different types of surgery confirmed no clinical advantage of OFA in pain control or reduction of opioid consumption. However, a recent meta-analysis, which adopted a more stringent OFA definition and, therefore, inclusion criteria, demonstrated lower opioid requirements in the first 24 h after the operation ^21^ and lower pain scores only in the first 2 h postoperatively.

Regarding the PONV rate, our study is in line with most studies^2,6,11,13,16–19^ and meta-analysis^15^ on OFA to show reduced incidence of PONV, to which opioid administration is the main factor. Avoiding opioids, even strictly intraoperatively, can positively impact the occurrence of PONV. However, there were differences in how long the OFA beneficial effect on PONV lasts. Mulier^2^ demonstrated a reduction of PONV rate not only in the close postoperative period as in our study but persisting to 24 h after the operation; a similar effect was described by Zimman-Giemmel et al.^13^, but in their study, the result was affected by the fact that only a single assessment was performed, making it difficult to precisely determine the effect of OFA use on the incidence of PONV over time. However, several studies also described a lack of difference in PONV incidence in the OFA group^12,14,18,22^.

In an attempt to explain the short-term benefits of the OFA on opioid consumption, NRS score, and PONV incidence in our study, we hypothesize that the observed differences are due to the limited duration of action of the coanalgesics used intraoperatively, such as dexmedetomidine or lidocaine, for which the half-life does not exceed 2 to 3 h, respectively ^23,24^. Hence, their effect is too short-lived to significantly affect the entire postoperative day.

To our knowledge, this is the first randomized trial in which patients in the OFA group for bariatric surgery showed significantly greater hemodynamic lability than in the MMA group, manifested by 40% greater ephedrine consumption and 20% greater crystalloid use. It can be expected as obese patients with comorbidities undergo laparoscopy in steep anti-Trendelenburg position, and they receive coanalgesics of which lidocaine, dexmedetomidine, and magnesium sulfate have hypotensive and cardio-depressive effects. Our study showed no significant difference in minimal BP values, but we explain this by immediate treatment of emerging hypotension. In correspondence to our findings, Soudi et al.^18^ demonstrated more frequent episodes of hypotension in the OFA group, which the authors explain by defining hypotension as a 20% decrease in BP from basal BP. On the contrary, no difference in BP values was shown in Mulier’s 2018 and Mansour’s 2013 study^2,20^. Still, the authors of these trials do not report the average dose of vasopressors nor the volume of fluids administered, which are crucial to assess the prevalence of hypotension requiring intervention. In contrast to our results, in a retrospective study performed by Berlier et al., patients in the OFA group with clonidine or dexmedetomidine required vasopressors less frequently than in an opioid-based group, whereas episodes of hypertension occurred more often^25^. However, the authors of the study acknowledge that there were several confounding factors and limitations, such as significant differences in anesthesia mode between the groups^26^, as well as methodological limitations in the anesthesia protocols used ^27^. In general surgery, aligning with our study, a significantly higher incidence of hypotension while using OFA was described by Helal et al.^28^, who demonstrated such a phenomenon in obese patients undergoing laparoscopic cholecystectomy.

There are safety concerns stemming from the study conducted by Beloil et al.^6^. The study had to be terminated after enrolling 312 patients because of significant hemodynamic instability in the OFA group. In a comparative study between OFA and opioid-based anesthesia utilizing remifentanil, five cases of severe bradycardia were observed, including one instance of asystole. On the contrary, in our trial, it was observed that the group receiving MMA treatment exhibited a reduction in their minimal HR. However, this observation did not result in any significant alterations in the administration of atropine. Potential factors contributing to the variance in outcomes may include the administration of higher dosages of dexmedetomidine, averaging 1.2 mcg/kg/hour, during a relatively prolonged anesthesia period, with an average duration of 268 min. It is also important to note that the study did not specifically focus on bariatric surgery.

Based on the research above, the potential for hemodynamic lability in individuals with OFA presents a substantial concern, as hypotension may lead to such consequences as myocardial injury or kidney failure^29^. This is particularly pertinent for those with ischemic heart disease, which is not uncommon in the obese, hypovolemia, or orthostatic hypotension^3,30^.

The effects of specific components of multimodal analgesia can vary depending on the procedure and may differ between types of surgeries^31^. The strength of our study is its practical relevance as it investigates the impact of OFA on patients who have undergone one specific type of operation LSG, which according to The International Federation for the Surgery of Obesity and Metabolic Disorders, is the most commonly performed bariatric surgery^32^. We strived for maximum objectivity during our assessment by utilizing PCA and monitoring pain scores, opioid doses, and PONV rates at fixed intervals.

Limitations of our study include the choice of remifentanil in the MMA group, which may increase pain intensity during the first 24 h^33^. In our selection, we adhered to the recommendation from the ERABS guidelines to use drugs with as short a half-life as possible in bariatric anesthesia^4^. Another limitation is the discontinuation of coanalgesic infusion in the OFA group at the end of surgery. The benefit to the patient could be more clinically relevant with the maintenance of these infusions. However, the safety and validity of such treatment require further study.

In conclusion, the mode of anesthesia did not influence pain scores or opioid administration after the operation. Moreover, the advantages of lessening the incidence and severity of PONV and reducing opioid use were only evident for a limited period following the surgery. Both forms of anesthesia allowed for patient discharge within a day of the procedure. Patients who received OFA required more interventions to maintain their hemodynamic stability, indicating the need for further research to assess its safety and efficacy.

## Data Availability

The data generated and analyzed during the current study are available from the corresponding author upon reasonable request.
